# Soil-transmitted helminths: A critical review of the impact of co-infections and implications for control and elimination

**DOI:** 10.1371/journal.pntd.0011496

**Published:** 2023-08-10

**Authors:** Sarah Lebu, Winnie Kibone, Chimdi C. Muoghalu, Stephen Ochaya, Aaron Salzberg, Felix Bongomin, Musa Manga

**Affiliations:** 1 The Water Institute at UNC, Department of Environmental Sciences and Engineering, Gillings School of Public Health, University of North Carolina at Chapel Hill, Chapel Hill, North Carolina, United States of America; 2 School of Medicine, College of Health Sciences, Makerere University, Kampala, Uganda; 3 Department of Microbiology and Immunology, Faculty of Medicine, Gulu University, Gulu, Uganda; 4 Department of Biology, Faculty of Science, Gulu University, Gulu, Uganda; 5 Department of Clinical Pathology, Uppsala Academic Hospital, Uppsala, Sweden; Federal University of Ceará, Fortaleza, Brazil, BRAZIL

## Abstract

Researchers have raised the possibility that soil-transmitted helminth (STH) infections might modify the host’s immune response against other systemic infections. STH infections can alter the immune response towards type 2 immunity that could then affect the likelihood and severity of other illnesses. However, the importance of co-infections is not completely understood, and the impact and direction of their effects vary considerably by infection. This review synthesizes evidence regarding the relevance of STH co-infections, the potential mechanisms that explain their effects, and how they might affect control and elimination efforts. According to the literature reviewed, there are both positive and negative effects associated with STH infections on other diseases such as malaria, human immunodeficiency virus (HIV), tuberculosis, gestational anemia, pediatric anemia, neglected tropical diseases (NTDs) like lymphatic filariasis, onchocerciasis, schistosomiasis, and trachoma, as well as Coronavirus Disease 2019 (COVID-19) and human papillomavirus (HPV). Studies typically describe how STHs can affect the immune system and promote increased susceptibility, survival, and persistence of the infection in the host by causing a TH2-dominated immune response. The co-infection of STH with other diseases has important implications for the development of treatment and control strategies. Eliminating parasites from a human host can be more challenging because the TH2-dominated immune response induced by STH infection can suppress the TH1 immune response required to control other infections, resulting in an increased pathogen load and more severe disease. Preventive chemotherapy and treatment are currently the most common approaches used for the control of STH infections, but these approaches alone may not be adequate to achieve elimination goals. Based on the conclusions drawn from this review, integrated approaches that combine drug administration with water, sanitation and hygiene (WASH) interventions, hygiene education, community engagement, and vaccines are most likely to succeed in interrupting the transmission of STH co-infections. Gaining a better understanding of the behavior and relevance of STH co-infections in the context of elimination efforts is an important intermediate step toward reducing the associated burden of disease.

## 1.0. Introduction

According to the World Health Organization (WHO), approximately 1.5 billion people are infected by soil-transmitted helminths (STHs) globally, with the highest prevalence reported from sub-Saharan Africa, South America, and Asia [1]. Infections are caused by 4 major species of nematodes, namely, the roundworm (*Ascaris lumbricoides*), the whipworm (*Trichuris trichiura*), the hookworms (*Ancylostoma duodenale* and *Necator americanus*), and the threadworm (*Strongyloides stercoralis*), often collectively referred to as geohelminths. Moderate and high-intensity infections produce clinical manifestations such as vomiting, diarrhea, abdominal pain, and weight loss [2]. The effects can be more devastating among children, malnourished, and immune-compromised individuals [2,3]. Particularly among school-going children, intestinal helminth infections have been reported to have long-term consequences such as micronutrient deficiency and iron deficiency [3]. Moreover, studies have reported increased incidences of stunting, cognitive growth retardation, school absenteeism, and poor academic performance associated with STH infections among children [4,5].

Risk factors for transmission of STH infections are poor sanitation conditions, inadequate water supply, overcrowding, lower socioeconomic status, living in proximity to animals, and rural areas more than urban areas [6]. Previous systematic reviews have reported an association between hygiene-related behavioral risk factors and the transmission of STH infections. For example, among schoolchildren, the following meta-analytic estimates have been reported: poor handwashing (OR 1.55, 95% CI 0.61 to 3.92, *p* < 0.001), open defecation (OR 5.20, 95% CI 1.35 to 20.16, *p* < 0.001), untrimmed fingernails (OR 3.21, 95% CI 1.57 to 6.55, *p* = 0.001), and not wearing footwear (OR 29.5, 95% CI 6.59 to 132.55, *p* < 0.001) [7,8]. Other adverse risk factors discussed in the literature include nail biting, soil eating, eating raw unwashed vegetables, swimming in water bodies, and close contact with animals [9–13].

STH parasites can interact with multiple other pathogens to cause co-infections and comorbidities in humans. The causes of co-infection are complex and multifactorial, and the evidence on the direction and magnitude of concurrent infections is varied [14]. The principal mode of action underlying co-infections is the ability of STHs to act as immunomodulators, meaning that they can affect the host’s immune response in ways that facilitate their survival and persistence in the host [14,15]. The immune response to STHs is characterized by a strong type 2 helper T-cell (TH2) response, which is associated with the production of cytokines such as interleukin-4 (IL-4), IL-5, and IL-13 [16,17]. This response is thought to be critical for the survival of STHs in the host, as it can suppress the type 1 helper T-cell (TH1) response required to control other infections. This immune response action works by invading cutaneous or mucosal sites and functions as protective immunity against other pathogens [17]. In addition, this type of immune response is associated with increased susceptibility to other infections, particularly viral and bacterial infections, as it does not provide strong protection against these types of pathogens [14].

On the other hand, some studies have suggested that STH infections may also have a protective effect against certain infections [2]. For example, a study in Cameroon found that children co-infected with STHs and malaria had a lower risk of severe anemia than those infected with malaria alone, implying that malaria and helminth co-infection was protective against anemia in that study context [18]. The TH2-dominated immune response induced by STHs may have a modulatory effect on the host’s immune response to other pathogens, reducing excessive inflammation and tissue damage [19].

The co-infection of STH and other infectious diseases has important implications for treatment and control strategies. Elimination of parasites from a human host can become much more difficult because the TH2-dominated immune response induced by STH infection can suppress the TH1 immune response required to control other infections, leading to increased pathogen load and disease severity [20]. Other mechanisms that make co-infections more challenging to eliminate include alteration of the performance of diagnostic tests, vaccine response, and drug–drug interactions during treatment [21].

Overall, the interaction between STH infections and other infectious diseases is complex, and more research is needed to fully understand the underlying mechanisms and the potential for co-infections to influence disease outcomes. Although there is substantial literature on the epidemiologic patterns of STH infections and their control, mechanisms, and the significance associated with co-infections with other diseases are still not well understood. The goal of the review is to synthesize existing evidence on the impact, etiology, and considerations for reduced transmission of STH co-infections with other infectious diseases of concern. Increasing the evidence of the relevance and behavior of STH co-infections in the context of elimination efforts is an intermediate step for reducing the associated burden of disease.

## 2.0. Methods

We searched PubMed, Scopus, and Google Scholar for studies that describe commonly reported co-infections of STHs and other diseases of significant public health burden [21]. The search was conducted in November 2022 and updated in March 2023. The scope of the search focused on STH infections with malaria and anemia, human immunodeficiency virus (HIV), tuberculosis, pregnancy-related anemia, pediatric anemia, neglected tropical diseases (NTDs) (lymphatic filariasis, onchocerciasis, schistosomiasis, trachoma), Coronavirus Disease 2019 (COVID-19), and human papillomavirus (HPV). We used different combinations of the following keywords in our search: “*soil-transmitted helminths and malaria co-infections*,” “*soil-transmitted helminths and anemia co-infections”* (with variations including *pregnant women* and *children*), “*soil-transmitted helminths and HIV co-infections*,” “*soil-transmitted helminths and tuberculosis co-infections*,” “*soil-transmitted helminths and COVID-19 co-infections*,” “*soil-transmitted helminths and HPV co-infections*,” “*soil-transmitted helminths and schistosomiasis co-infections*,” “*soil-transmitted helminths and W*. *bancrofti co-infections*,” “*soil-transmitted helminths and T*. *solium co-infections*,” “*soil-transmitted helminths and T*. *cruzi co-infections*,” *and* “*soil-transmitted helminths and onchocerciasis co-infections*.” We also searched reference lists from the identified studies. The inclusion criteria were predefined as studies providing information on STH co-infections with other diseases (including prevalence, host–pathogen interactions, biological mechanisms of action, and control strategies). Original field studies, reviews, models, laboratory-based studies, viewpoints, and letters to the editor were included. Studies published in a language other than English and gray literature were excluded from the review. In addition, articles were excluded if they focused on STH infections alone, rather than as co-infections alongside other diseases. However, we did not apply exclusion criteria with regards to the date of publication of the study.

Based on these criteria, 106 relevant studies were identified and used to synthesize information on the burden of STH co-infections, relevance, mechanisms of immunoregulatory action, and implication on control of infections. A flowchart documenting article screening and reasons for exclusion is provided in S1 Fig. As the impact and control of STH co-infections is a relatively understudied area, we opted not to further screen the identified studies. This is because a more inclusive approach to the available literature could provide a more detailed and thorough comprehension of the limited state of knowledge in the field. Table 1 provides an overview of the synthesis matrix that was used to organize and synthesize the research findings. Box 1 summarizes the key findings from the review. In Box 2, we highlight a set of 5 crucial papers in the field that demonstrate the evidence regarding the significance of STH co-infections, the history of their control and elimination, and the future strategies to consider for their control and elimination.

**Table 1:** Mechanisms of immunological response and implications for control and elimination of STH co-infections with other diseases.

Coinfecting disease	Outcome	The direction of association*	Hypothetical immunological mechanisms	Implications for control	Reference(s)
Malaria and malaria-induced anemia	*Ascaris* have been associated with reduced incidence, prevalence, or parasitemia	Negative	I. Immunomodulation generated by both parasites may indicate a cross-reactivity between helminths and plasmodia that could confer protection to co-infected subjects. II. Inhibition of dendritic cell maturation by *P*. *falciparum*-infected erythrocytes. III. Genetic susceptibility/resistance of the host.	Single antihelminthic community therapy programs targeting *Ascaris* could prevent an increase in malaria incidence.	[23,24]
	Hookworms have been associated with increased malaria incidence and severity.	Positive	I. T cells with a regulatory function may be preferably induced in helminth-infected patients thereby leading to a suppression of TH1 cells and proinflammatory activity. I. Hookworm-related blood loss may boost cues attractive for the vector such as increased respiratory and cardiac output thus leading to a greater probability of infective bites.	Significant progress has been achieved in the development of vaccines against hookworms, and ongoing medical studies are exploring vaccines that focus on the infectious L3 stage larvae and adult worms.	[28,85]
	*Ascaris* co-infection neither exacerbates nor ameliorates the severity of malarial anemia.	No association	I. Helminth-induced immunoregulatory cytokines may reduce the magnitude of the proinflammatory immune response induced by malaria infection, but there may not be any visible effect among asymptomatic people because of a prevailing malaria-induced immunoregulatory response.	Ensuring adequate access to diagnosis and treatment of malaria is paramount in protecting co-infected people from the devastating effects of severe malaria-induced anemia.	[24,25]
HIV	Helminth co-infection in HIV-1–infected individuals may result in increased plasma levels of HIV-1 RNA. Helminth infections may reduce the number of CD4+ cells.	Positive	I. Persistent antigenic stimulation may cause abnormal activation of the immune system, resulting in peripheral lymphocytes that are susceptible to HIV infection. II. Immunoregulation from helminth infections may suppress HIV-1-specific CD4 and CD8 counts and cytokine production, which may compromise control of HIV-1 replication.	Deworming with antihelminthics may slow the progression of HIV-1. Treating confirmed helminth infections in HIV–positive adults showed a small suppressive effect on mean plasma viral load.	[30,32–34]
Tuberculosis	Increase in tuberculosis morbidity and mortality.	Positive	I. Infection from STH can cause activation of latent tuberculosis infection and suppresses both innate and adaptive immunity. II. Co-infection with *S*. *stercoralis* can reduce the activation of antigen-stimulated type 1 and type 17 cytokines, promoting an increase in systemic type 2 and regulatory cytokines.	Co-infections are reversible (for the most part) by antihelminthic treatment.	[40–43]
Gestational anemia	The risk of anemia increases with increasing intensity of either *Trichuris* infection or hookworm infection, and hookworm infection is associated with a decrease in blood hemoglobin.	Positive	I. During pregnancy, STH infections can increase systemic inflammation that subsequently disrupts placental vasculogenesis and angiogenesis, and in turn, negatively impact fetal growth and development.	WHO recommends a single dose of preventive chemotherapy administered during the first trimester in areas with a high baseline prevalence of helminth infection. Concerns about safety and potential side effects should be considered.	[44–46]
Pediatric anemia	STH infections are associated with a higher risk for anemia among children.	Positive	I. STH infections can disrupt the normal function of the intestinal epithelium, leading to malabsorption of iron, chronic blood loss, and intestinal inflammation.	It is recommended that both STH detection and hemoglobin level assessment be conducted simultaneously in school-aged children.	[52,53,86]
NTDs	Co-infections with lymphatic filariasis, onchocerciasis, schistosomiasis, and trachoma commonly occur in co-endemic areas.	Inconclusive	Not available	Innovative and integrated control programs such as improving household-level conditions, access to improved water and sanitation, intensive disease management; vector control, bed nets, and vaccines.	[23,62,63,69,87,88]
COVID-19	COVID-19 may be less severe in patients with preexisting helminth infections. STH treatment can decrease the morbidity, hospitalization rate, and mortality associated with COVID-19. However, helminth-induced immunosuppression activity could be disadvantageous by reducing vaccination efficacy.	Negative	I. Through an excessive immune response and subsequent cytokine storm, helminth-driven immune modulation can contribute to the less severe outcomes of COVID-19. II. Expansion of Treg cells coupled with modulation of monocyte/macrophage trafficking and activation. III. Infections can suppress the immune responses and mitigate SARS-CoV-2 vaccine efficiency among people who have been vaccinated.	Further studies are warranted in a cohort of SARS-CoV-2-infected individuals residing in helminth and air pollution endemic regions to offer more clarification.	[72,75,76]
HPV	Increased HPV prevalence associated with STH infections.	Positive	I. Excess in detectable levels of HPV might be explained by a Th2-biased mucosal immune response secondary to STH infection.	Consideration of antiviral treatment for HPV alongside helminth deworming programs.	[79–81]

* This list includes only predominant associations of co-infections as identified in the literature. This list is not exhaustive.

## 3.0. Co-infection with other diseases: Burden, etiology, and implications for control

### 3.1. Malaria and malaria-induced anemia

The body of literature on STH-malaria co-infections can be synthesized into broad trends. Studies have found a positive association between malaria and STH co-infections. For example, in a systematic review conducted on 22,114 children in 13 countries, a pooled estimate showed a prevalence of plasmodium-helminth co-infections of 17.7% (95% CI 12.7 to 23.2) [22]. This study also reported that the odds of anemia were higher in children co-infected with malaria and STH than in children with malaria infection alone (OR 1.20, 95% CI 0.59 to 2.45). *A*. *lumbricoides* have an overall protective effect on malaria (incidence, prevalence, or reduction of parasitemia) [23,24]. Studies that demonstrate a clear negative interaction between *A*. *lumbricoides* and *P*. *falciparum* density have an important implication for treatment; single antihelminthic community therapy programs targeting *Ascaris* could prevent an increase in malaria incidence [23]. However, there is scarce literature on the effect of *Ascaris*-malaria co-infections with contradictory results. Some studies have reported increased or no association with *P*. *falciparum* parasitemia or malaria-induced anemia among people infected with *A*. *lumbricoides* [25–27]. Hookworms have been associated with increased malaria incidence and parasitemia. For example, a systematic review conducted in sub-Saharan Africa reported that 45.1 million (95% CI 43.9 to 46) (25%) school-aged children are at a coincidental risk of hookworm and malaria infection risk [28]. Similar studies have also confirmed that malaria parasites are more prevalent in hookworm-infected children than in hookworm-free children [29]. Further, *P*. *falciparum* and STH infections have long been recognized as major contributors to anemia as well as other complications of malnutrition, especially in malaria-endemic countries. A study reported that the odds ratio of having anemia among adults infected with both malaria and STH was 2.91 (95% CI 1.38 to 6.14) while the odds ratio of the association between anemia and malaria alone was 1.53 (95% CI 0.97 to 2.42) [29]. One hypothesis that describes the nature of interactions between malaria and hookworm infections could be linked to the combination of immune modulation and hookworm-related blood loss, which might increase cues attractive for the vector such as increased respiratory and cardiac output thus leading to a greater probability of infective bites [24]. On the other hand, the apparent decrease of malaria in *Ascaris* infections can be explained by the regulatory function of T cells preferably induced in helminth-infected patients thereby leading to a suppression of TH1 cells and proinflammatory activity [24]. Significant progress has been achieved in the development of vaccines against hookworms, and ongoing medical studies are exploring vaccines that target the infectious larvae and adult hookworms at the L3 stage.

### 3.2. Human immunodeficiency virus (HIV)

Research examining the prevalence of detectable helminth infections among individuals who are HIV-1 seropositive has reported a prevalence range of 12.5% to 19.3% [30,31]. There is insufficient evidence to conclusively determine the potential benefit of helminth eradication in HIV-1 and helminth co-infected adults. Current evidence suggests that helminth co-infection in HIV-1–infected individuals may result in increased plasma levels of HIV-1 RNA, and therefore, deworming seropositive patients with antihelminthics could possibly slow the progression of the HIV-1 disease [32,33]. In several studies, treating confirmed helminth infections in HIV–positive adults showed a small suppressive effect on mean plasma viral load at 6 to 12 weeks following deworming (difference in mean change −0.13 log10 viral RNA, 95% CI 0.26 to 0.00, *p* = 0.04) [32] and −0.54 log10 viral HIV-1 RNA; *p* = 0.09) [34], suggesting that deworming may be an important potential strategy to delay HIV-1 progression. However, the evidence on the association of CD4 count with persistent helminth infection is varied, with some studies finding no significant association [30] and others reporting significant increases [34,35] among HIV–positive individuals treated with antihelminthics. Several factors have been implicated in facilitating the progression of HIV-1 among individuals with STH infections. First, persistent antigenic stimulation may cause abnormal activation of the immune system, resulting in peripheral lymphocytes that are susceptible to HIV infection [36]. Second, immunoregulation from helminth infections may suppress HIV-1–specific CD4 and CD8 counts, and cytokine production, which may compromise control of HIV-1 replication [37]. Another hypothesis is that chronic helminth infections are associated with antigen-specific anergy and hyporesponsiveness that have the potential to down-regulate control of HIV-1 replication [38]. Finally, immune activation can trigger a cellular response that manifests in susceptibility to HIV-1 infection [39]. In conclusion, while the evidence is still limited and mixed, deworming with antihelminthics may be a potential strategy to delay HIV-1 progression in individuals co-infected with helminths. Further research is needed to establish the effectiveness of this strategy and to explore the underlying mechanisms of the association between helminth infections and HIV-1 progression.

### 3.3. Tuberculosis

STH co-infection with tuberculosis may cause activation of latent tuberculosis infection and suppress both innate and adaptive immunity, leading to an eventual increase in morbidity and mortality [40]. In a cross-sectional study, the prevalence of intestinal parasite co-infections among tuberculosis patients was reported as 16.1% [41]. A systematic review estimated a higher pooled prevalence of 33% (95% CI: 23.3, 44.3), with the most common parasites being *A*. *lumbricoides* at 10.5% (95% CI: 6.0, 17.5), followed by hookworm at 9.5% (95% CI: 6.10, 14.4), and *Strongyloides sterocoralis* at 5.6% (95% CI: 3.3, 9.5) [42]. It is estimated that patients with tuberculosis are twice as likely to be infested with intestinal helminths and are 2 to 3 times more likely to harbor ≥1 intestinal parasite compared to people without tuberculosis [42]. However, real-world evidence that helminth–tuberculosis interactions are clinically important is less convincing [21]. In latent tuberculosis, co-infection with S. *stercoralis* can reduce the activation of antigen-stimulated type 1 and type 17 cytokines, and instead, promote an increase in systemic type 2 and regulatory cytokines [21,43]. Note that protection from tuberculosis requires a type 1 helper T cell (Th1) response. In a study in Ethiopia, among HIV–positive patients who have tuberculosis, administering the HAART (highly active antiretroviral therapy) might have contributed to the rapid decline in worm rate seen in the study without direct antihelminthic therapy [41]. In this study, as a result of tuberculosis treatment, the worm infection rate of HIV and tuberculosis-infected patients declined from 31% at week 0 to 9% at week 2 of treatment, whereas in nonpatients, the worm infection rate showed no change [41]. Altogether, literature on STH-tuberculosis infections seems to suggest that co-infected individuals might be at an increased risk for tuberculosis morbidity and mortality, particularly for latent tuberculosis cases, but the real-world evidence is less convincing.

### 3.4. Pregnancy-related anemia

Pregnancy-induced anemia facilitated by STH infections is of particular concern because of its association with maternal mortality. Previous studies have estimated that gestational anemia is common in developing countries, affecting approximately 57% of pregnancies [44], and the prevalence of STH infections during pregnancy range from 11% to 31% [45]. The association between hookworm infection and anemia in pregnancy is well demonstrated. In a meta-analysis conducted in 2008, a hookworm infection of 1,999 eggs per gram was found to be associated with a significant decrease in blood hemoglobin [46]. There is limited evidence demonstrating that, in cases where there is hookworm infection, the risk of anemia increases [47]. During pregnancy, hookworm infections can increase systemic inflammation that subsequently disrupts placental vasculogenesis and angiogenesis, and in turn, negatively impact fetal growth and development [46]. For treatment, WHO recommends preventive chemotherapy administered to pregnant women as a single dose of either 400 mg of albendazole or 500 mg of mebendazole during the first trimester in areas with a high baseline prevalence of helminth infection (20%) and anemia (40%) [46]. The effects of preventive chemotherapy have been studied among pregnant women and have shown a reduction of the risk of maternal anemia in the third trimester (RR 0.94, CI 0.81 to 1.10) [48]. Overall, controlling STH infections during pregnancy is crucial for maternal and fetal health, and preventive chemotherapy can be an effective strategy to reduce the burden of gestational anemia in areas with a high prevalence of STH infections. Future research and programs may focus on further understanding and dispelling potential side effects of routine antihelminthic treatment during pregnancy.

### 3.5. Pediatric anemia

Studies have reported that STH infections are associated with a higher risk of anemia among children. For example, in Ethiopia, children infected with intestinal helminths were highly likely to have anemia (aOR 8.87, 95% CI 2.28 to 34.58) [49]. A meta-analytical study confirmed the direction of this effect by reporting a high OR 4.49, 95% CI 1.58 to 12.75, *p* < 0.05 among children with multiple STH infections [50]. The study delineated the effect of single STH parasites on child anemia and reported the greatest risk of anemia among hookworm-infected children with OR 3.3, 95% CI 1.98 to 5.49, *p* < 0.05, while *A*. *lumbricoides* had OR 1.57, 95% CI 1.2 to 2.07, *p* < 0.05 and *T*. *trichiura* with OR 1.66, 95% CI 1.13 to 2.43, *p* < 0.05 [50]. These results confirm the hypothesis that among children who are infected with STH, the mean hemoglobin levels are significantly lower in individuals with polyparasitism (referring to individuals harboring more than 1 helminth species in their body) [51]. Contrary results have been reported in Indonesia, with a single-species STH infection being associated with a lower risk of anemia (OR 0.320, 95% CI 0.126 to 0.809, *p* = 0.016) [52]. One hypothesis for this effect is that STHs can cause malabsorption of nutrients like iron, by damaging the intestinal wall [53]. The worms can disrupt the normal function of the intestinal epithelium, leading to the malabsorption of iron and other nutrients, which can exacerbate iron deficiency and contribute to the development of malaria. In addition, STHs attach to the intestinal wall and feed on the host’s blood and other nutrients, leading to chronic blood loss and intestinal inflammation [54]. The evidence suggests that there is a clear correlation between STH infections and an increased risk of anemia among children. This relationship is particularly significant in cases where hookworm is present. Based on these findings, it is recommended that both STH detection and hemoglobin level assessment be conducted simultaneously among school-aged children.

### 3.6. Neglected tropical diseases (NTDs)

STHs may be endemic in settings with a high burden of other NTDs, such as schistosomiasis, trypanosomiasis, onchocerciasis, and others. A framework for morbidity control and the potential elimination of NTDs was formulated in the London Declaration of 2012, to control lymphatic filariasis, onchocerciasis, schistosomiasis, trachoma, and STH infections through controlled chemotherapy [55]. Co-endemicity of schistosomiasis and STH infections has been reported in many developing countries, with the prevalence of co-infection among the general population ranging from 0.4% to 2.2% [56–59]. Hygiene-related risk factors that are associated with STH infections such as poor sanitation conditions, inadequate access to clean water, overcrowding, handwashing, shoe wearing, and nail trimming are simultaneously implicated with schistosomiasis infections [60]. STH and schistosomiasis infections can both be controlled through the periodic administration of preventive chemotherapy with albendazole and praziquantel, respectively [61]. School-aged children are the most affected risk profile, and therefore, most control programs target this age group. Important to note is that the burden of hookworm and *Strongyloides* infections tend to be higher among adults, e.g., those who live and work in agricultural settings, thus, treatment strategies that focus on school children may not be effective for at-risk adults [61]. Innovative variations of control programs have since been proposed to curb co-infections, such as: community-directed treatment and pediatric formulations of ivermectin for controlling onchocerciasis and STH infections among adults and children, respectively [62,63]; improving household-level conditions such as access to improved water and sanitation [60,64,65]; intensive disease management; vector control [66]; bed nets [67]; and vaccines [68,69]. Although challenges remain, control and elimination prospects for STH co-infections with other NTDs reflect the need for integrated and intimately linked intervention strategies focused on providing improved access to basic services and healthy hygiene behavior.

### 3.7. COVID-19

Recent studies on the association between COVID-19 and STH infections report mixed results. Helminth-induced immune regulation is both beneficial and detrimental to COVID-19 patients [70]. A study conducted in Ethiopia enrolled a cohort of COVID-19 patients and followed them progressively to assess the rate of STH infections among them [71]. They reported an STH infection rate of 33.8% among the patients and that COVID-19 was less severe in patients with preexisting helminth infections. In addition, COVID-19 patients with helminth co-infection had a lower probability of developing severe COVID-19 (aOR 0.37, 95% CI 0.17 to 0.80, *p* = 0.011) compared with those without helminth co-infection [71]. These results are consistent with other studies that reported negative associations between COVID-19 and helminth co-infections in helminth-endemic regions with aOR 0.23, 95% CI 0.17 to 0.30, *p* < 0.0001 [71–73]. The pathology of COVID-19 is primarily mediated by an excessive immune response and subsequent cytokine storm, thus helminth-driven immune modulation will hypothetically contribute to less severe outcomes of COVID-19 [74]. Emerging reports also stated that helminth co-infections can have a protective effect on COVID-19 patients via two mechanisms of action. First, STH treatment can decrease the morbidity, hospitalization, and mortality rates associated with COVID-19 through the expansion of Treg cells coupled with modulation of monocyte/macrophage trafficking and activation [72,75,76]. Secondly, researchers have shown that it is likely that people with helminth infections and type 2 immune responses might have a higher risk for both non-IgE-mediated and IgE-mediated anaphylaxis upon receiving the first vaccine dose and or booster doses, respectively [77]. As the literature on COVID-19 is evolving, more research should focus on understanding underlying immunology and consequences for joint SARS-CoV-2 vaccination and STH treatment [71]. In summary, research on COVID-19 and simultaneous infections with STH indicates that using antihelminthic treatment could potentially reduce the severity of COVID-19 and related complications. However, individuals with helminth infections might have an increased risk of experiencing anaphylaxis after getting vaccinated for COVID-19. As more studies are conducted, it is crucial to explore the immunological mechanisms involved and the implications for administering both the SARS-CoV-2 vaccine and STH treatment concurrently.

### 3.8. Human papillomavirus (HPV)

**Fig 1:**
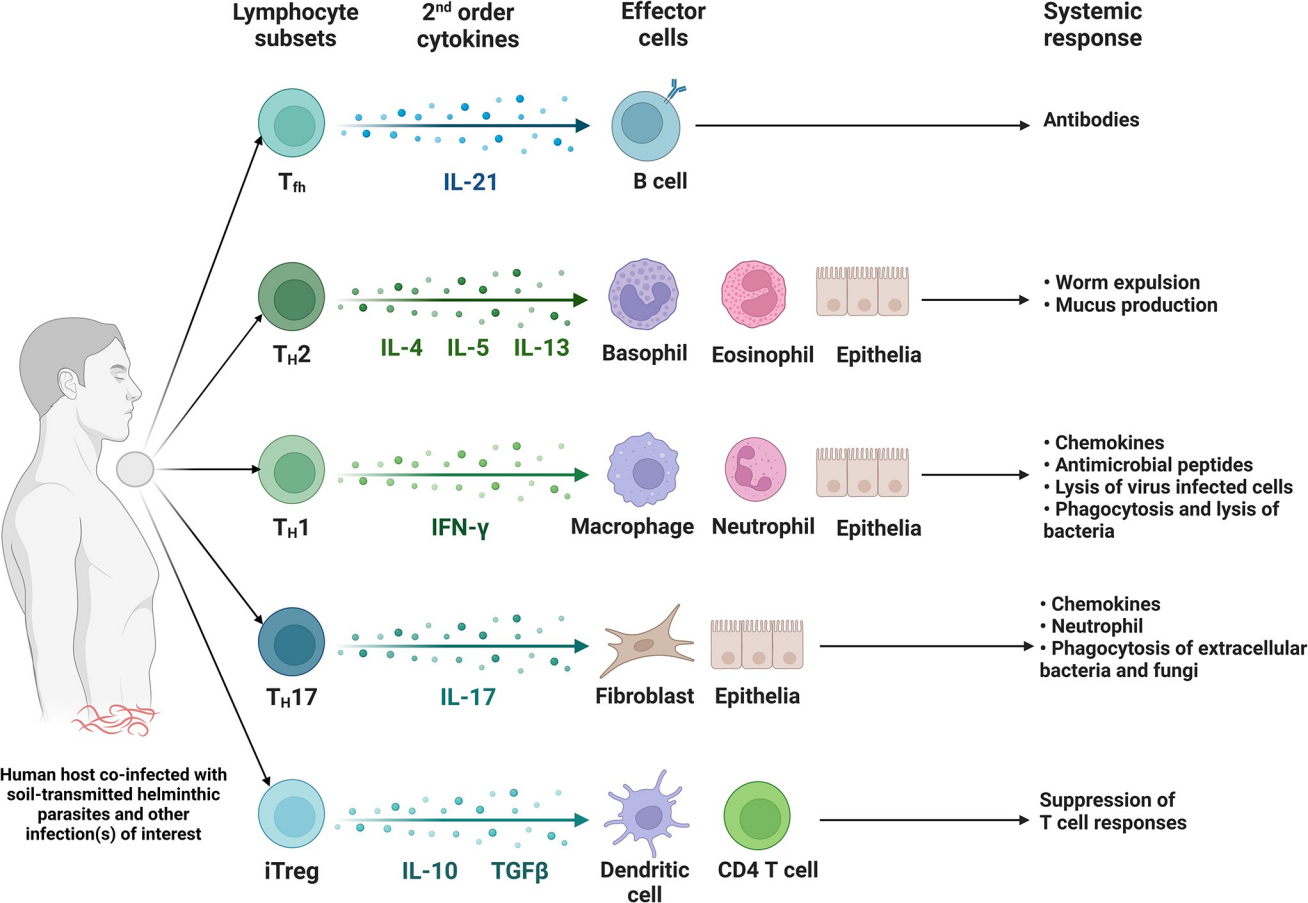
STHs employ both immune protection and immune suppression mechanisms to evade the host’s immune system. The induction of type 2 immune response is thought to be a crucial immune protection mechanism, while the suppression of dendritic cell function and T cell activation are key immune suppression mechanisms. Helminths trigger the Th2 response that activates cells to release anti-inflammatory and regulatory cytokines (e.g., IL-4, IL-5, IL-9, IL-10, IL-13, TGF-B). These cytokines mediate the activation of effector mechanisms that include the antibody-based immune response and regulatory T cells, inhibit the proinflammatory Th1 response, and modulate antigen activity [82–84]. Figure developed through www.biorender.com. STH, soil-transmitted helminth.

Studies have found an association between HPV, the primary cause of cervical cancer, and STH infection. A recent study conducted among women in Peru revealed that those with STH infection exhibited a 60% higher prevalence of HPV compared to their counterparts without STH infection (PR, 1.6; 95% confidence interval, 1.0 to 2.7). The hypothesis is that the excess in detectable levels of HPV might be explained by a Th2-biased mucosal immune response secondary to STH infection. This hypothesis is supported experimentally by a study conducted among women in Latin America, which reported a 1.6-fold increase in HPV prevalence [78]. More specifically, Th2-induced immunity in the female reproductive tract either via type 2 cytokines activating M2 macrophages or the differentiation of CD4+ T cells can boost type 1 immunity and increase susceptibility to HPV as illustrated in **Fig 1** [79]. Accordingly, researchers argue that patients infected with STH should be included in cancer vaccine studies [78]. Interestingly, exposure to hookworms may have a protective effect on cervical cancer cell progression [80]. This effect was demonstrated by a laboratory experiment with rodent models of human hookworm whose findings show the role of altering epithelial–mesenchymal transition (EMT) marker expression and a selective inhibitory effect on cervical cancer cell migration [80]. Contradicting findings reported hookworm infection as inducing type 1/type 2 immune signature in the reproductive female tract of women who tested positive for HPV [81]. These findings highlight STH infection as a significant risk factor for acquiring HPV and potentially raise the need to consider antiviral treatment alongside helminth deworming programs.

Table 1 summarizes STH co-infections with the diseases discussed above, including plausible biological mechanisms of action and considerations for advancing control and elimination.

## 4.0. Rethinking elimination strategies for STH co-infections

Efforts targeting the control of STH co-infections have focused on antihelminthic treatment through the large-scale administration of albendazole or mebendazole deworming medication. Most programs have targeted school-aged children through school-based delivery schemes. This approach is considered cost effective and appropriate because children have the highest infection rates and can be reached easily through schools. In 2018, a total of 576 million (59.9%) of the estimated 1.1 billion children requiring deworming medication received treatment and this number is expected to continue rising [89]. Despite its contribution to controlling STH morbidity, targeted drug administration alone appears unsuccessful at eliminating STH infections due to the resurgence of reinfections, limited efficacy of current drugs, gaps in drug availability, and the risk of antihelminthic drug resistance [64].

Recent research has pointed to the importance of expansion of STH elimination strategies beyond mass drug administration in schools to additional subpopulations at risk (e.g., in humanitarian settings, prisons, fishing communities) and through water, sanitation, and hygiene (WASH) interventions, public education, and community engagement efforts [2,90]. Although current WHO goals emphasize morbidity control, WASH interventions have been proven to successfully interrupt STH transmission, although the evidence on effect estimates is mixed [91,92]. A systematic review on the effect of WASH on STH infections reported a protective effect from using treated water (OR: 0.46, 95% CI 0.36 to 0.60), access to improved sanitation (OR: 0.66, 95% CI 0.57 to 0.76), handwashing after defecating (OR: 0.45, 95% CI 0.35 to 0.58), and wearing shoes (OR: 0.30, 95% CI 0.11 to 0.83) [93]. The impact of WASH in effectively eliminating STH can be influenced by coverage, adherence, sustained usage, and quality of the intervention [94].

Health education programs that focus on teaching best hygiene practices and sanitary behaviors are often based on the premise that knowledge would be increased and health-promoting behaviors against increasing STH infections would be executed. These programs have been commonly implemented among school children, particularly because they tend to adapt and change behaviors at a young age [64]. Successful reduction of STH prevalence has been noted in multiple programs, implemented as stand-alone strategies or alongside mass drug administration [95–97].

Vaccines are considered an effective long-term solution to control and eliminate STH infections. Advances have been made in the development of vaccines against STH through animal models, but there is sparse data regarding understanding vaccine-elicited immune responses in human hosts [98]. Moreover, STHs have complex genomes and proteomes that make it difficult to identify antigenic targets for the development of an effective vaccine. Nonetheless, preclinical vaccine trials have identified potential candidates for hookworm [99], *Ascaris* [100,101], and *T*. *trichuriasis* [102] vaccines. Vaccine development efforts for STHs have primarily focused on the development of recombinant protein-based vaccines, as well as DNA-based and live attenuated vaccines. One of the most promising recombinant protein-based vaccine candidates is Na-GST-1, which is based on the glutathione S-transferase enzyme found in the intestinal tissue of hookworms [103,104]. Clinical trials have shown that Na-GST-1 may decrease fecal egg count and clinical pathology associated with hookworms [103]. Another promising recombinant protein-based vaccine candidate is *Ancylostoma ceylanicum* metalloprotease-1 (Ace-MTP-1), which has shown efficacy in preclinical studies against hookworm infections [104]. Another vaccine candidate is Tm-WAP49 protein, a recombinant protein vaccine against *T*. *trichiura*. A Phase 1 clinical trial found that the vaccine was safe and induced an immune response in vaccinated individuals [105]. Further studies are ongoing to assess the vaccine’s efficacy. In addition to recombinant protein-based vaccines, DNA-based vaccines have also been developed for STHs [106]. For example, a DNA vaccine targeting a protein found in the excretory-secretory products of hookworms has been shown to elicit protective immune responses in preclinical studies [107]. Live attenuated vaccines, which are weakened forms of the parasite, have also been investigated for STHs. One example is the irradiated hookworm vaccine, which has shown efficacy in animal models and has been evaluated in clinical trials [108]. While progress has been made in the development of STH vaccines, there is currently no approved STH vaccine due to challenges of vaccine efficacy, delivery, and cost effectiveness. Nonetheless, the development of effective vaccines against STHs remains a priority in global health efforts to control and eliminate these infections. Future research should focus on advancing clinical developments of vaccines and evaluating their efficacy and safety. Furthermore, studies should examine the efficacy of a cocktail vaccine containing multiple recombinant antigens against STH species and other infections.

**Fig 2:**
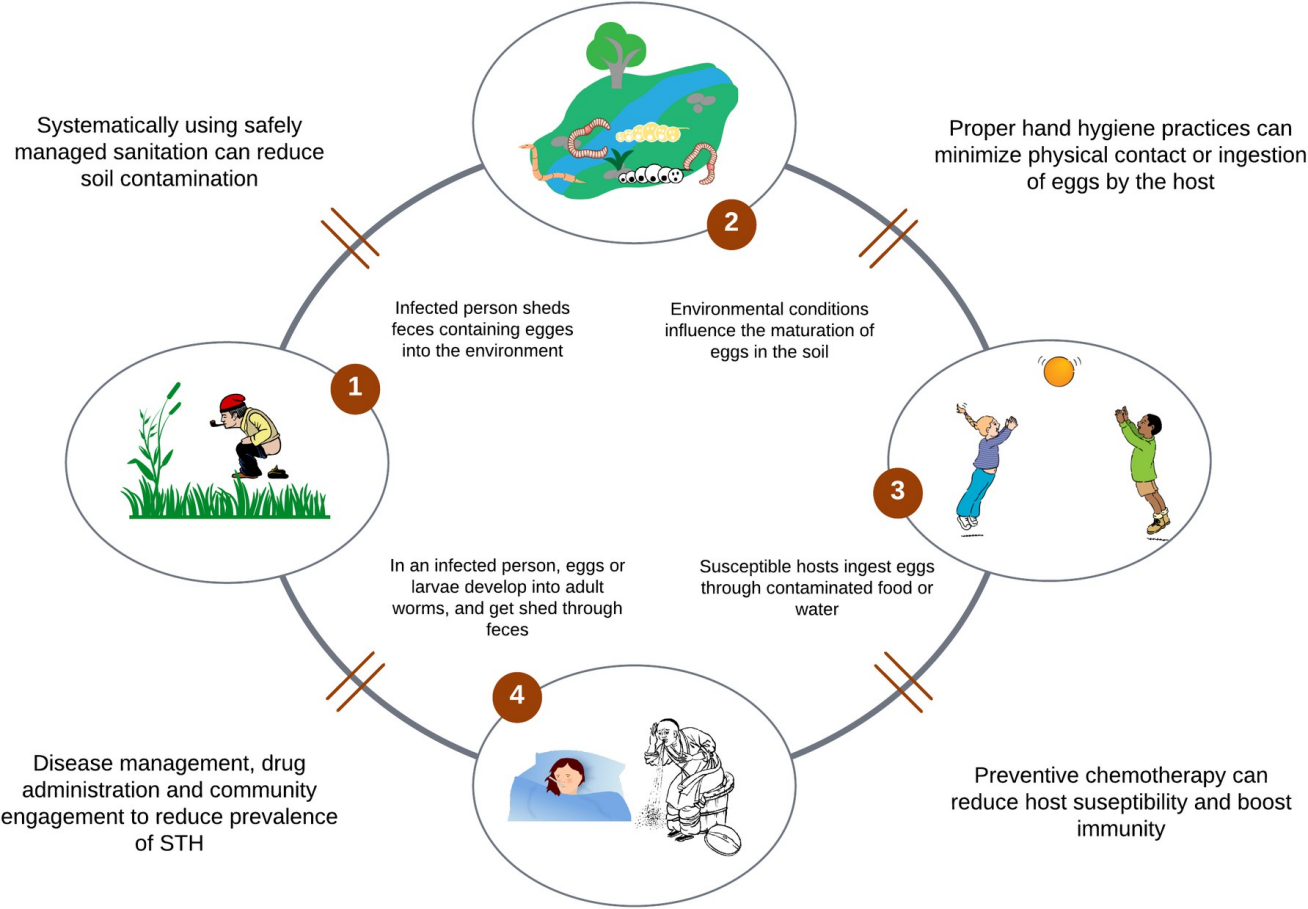
Integrated strategies for reducing the transmission of STH infections. Created by authors. STH, soil-transmitted helminth.

Several factors need to be considered for the optimal impact of control strategies:

1. **Examining immunomodulation caused by STH infections:** Due to the shared endemicity of pathogens in many geographical areas, co-infections between STH and other diseases are likely. An understanding of the immunomodulation mechanism of STH and resulting antagonistic and synergistic effects can inform intervention efforts. It is prudent to conduct more research on the mode of action of existing treatment regimen and their pharmacokinetic and pharmacodynamic effects in human hosts to fill the knowledge gaps in STH control [109]. Further, there is a need to identify and estimate the development of drug resistance mechanisms in STH control.
2. **Acknowledging the limitations of preventive chemotherapy in the fight against STH co-infections:** Overall, data shows that preventive chemotherapy can reduce the prevalence of STH but may unlikely be adequate to achieve the elimination of infections from all at-risk populations. Questions such as “who should be prioritized for treatment,” “how often should they be treated,” and “how long should the treatment be,” remain fully unanswered, particularly in cases of STH co-infections. More needs to be done if treatment is to be interrupted. For example, conclusions drawn from many studies postulate that treatment coverage should be wide and encompass all infected cases, the fidelity of the program should be high, and treatment should be more frequent and sustained over a long period if the transmission is to be interrupted by mass chemotherapy alone [61,110].
3. **Optimizing control efforts through integrated interventions:** A concerted effort of intervention strategies that incorporate WASH interventions, health education, and vaccines can effectively complement mass chemotherapy. National control programs can benefit from setting up coordinating committees to synchronize interventions and optimize overlapping areas. Fig 2 shows multiple transmission pathways for STHs and interventions that can be interrupted by the implementation of targeted, multifaceted interventions.
4. **Expanding antihelminthic treatment beyond school children subpopulations**: Exposure to STH is associated with increasing age, peaking among children aged 9 to 12 years [61]; hence, school children are usually the sentinel group for which interventions for antihelminthic treatments are implemented [64]. Differentially high prevalence has been reported among pregnant women, the elderly population, farmers, fishermen, and other at-risk populations, for whom control interventions are uncommon [64]. Besides, the burdens of hookworm and *Strongyloides* infection tend to be higher in adults than among children. To maximize health benefits to communities facing the high burden of co-infection, care must be taken to diversify control programs to reach all at-risk populations beyond school-aged children. Future research and intervention programs should target other age groups.

The main limitation of this review is that a limited number of infections that occur alongside STH infections were reviewed. Only infections that are commonly reported in the literature were discussed. This potentially contributes to the underreporting of rare co-infections between STH and other pathogens. More studies should exhaustively investigate understudied STH co-infections and their implications on control and elimination. Moreover, there is a need for more observational studies and meta-analytical studies to estimate the prevalence of STH co-infections among different populations.

## 5.0. Conclusions

Even though evidence of the effectiveness of existing STH elimination strategies is fairly understood, co-infections and comorbidities with other diseases pose a bigger challenge for elimination. This study highlights the high burden of disease caused by STH co-infections, underscoring the need for a more refined and coordinated delivery of interventions to achieve the WHO target of eliminating STH infections in highly endemic countries. A crucial aspect in the control of these co-infections is the ability of STH to act as immunomodulators on the human host’s immune system, resulting in synergistic or antagonistic effects that modify the severity of other diseases. Knowledge of the mechanisms of STH co-infections and immune response will help to design effective control strategies. Preventive chemotherapy and treatment are currently the most common approaches used for the control of STH infections, but these approaches alone may not adequately achieve elimination goals. Besides, there is little concerted effort in using antihelminthic treatment to strategically target co-infections with other diseases. Approaches that combine drug administration with WASH interventions, hygiene education, and community engagement have the greatest chance of interrupting the transmission of STH co-infections. Although progress has been made on nonclinical trials of vaccines against STH, more research should be done to understand their effectiveness in humans. Findings can inform policy and practitioner efforts toward controlling co-infections of STH and other diseases.

**Box 1. Key learning points**

1. There is substantial literature on the epidemiologic patterns of STH infections and their control; however, the significance and the mechanisms associated with co-infections with other diseases are still not well understood.
2. Immune response triggered by STH infections is characterized by a strong type 2 helper T-cell response.
3. This immune response is thought to be critical for immunomodulation in a human host, as it can suppress the type 1 helper T-cell response required to control other infections, thus, increasing the likelihood of co-infection.
4. Preventive chemotherapy alone can reduce the prevalence of STH but may unlikely be adequate for interrupting transmission and achieving elimination goals.
5. A concerted effort of intervention strategies that incorporate WASH interventions, health education, and vaccines can effectively complement mass chemotherapy.

**Box 2. Five key papers in the field**

1. Freeman MC, Akogun O, Belizario V Jr, Brooker SJ, Gyorkos TW, Imtiaz R, et al. Challenges and opportunities for control and elimination of soil-transmitted helminth infection beyond 2020. PLoS Negl Trop Dis. 2019 Apr 11;13(4):e0007201.
2. Banda GT, Deribe K, Davey G. How can we better integrate the prevention, treatment, control and elimination of neglected tropical diseases with other health interventions? A systematic review. BMJ Glob Health. 2021 Oct 1;6(10):e006968.
3. Montresor A, Mupfasoni D, Mikhailov A, Mwinzi P, Lucianez A, Jamsheed M, et al. The global progress of soil-transmitted helminthiases control in 2020 and World Health Organization targets for 2030. PLoS Negl Trop Dis. 2020 Aug 10;14(8):e0008505.
4. McArdle AJ, Turkova A, Cunnington AJ. When do co-infections matter? Curr Opin Infect Dis. 2018 Jun;31(3):209.
5. Vlas SJ de, Stolk WA, Rutte EA le, Hontelez JAC, Bakker R, Blok DJ, et al. Concerted Efforts to Control or Eliminate Neglected Tropical Diseases: How Much Health Will Be Gained? PLoS Negl Trop Dis. 2016 Feb 18;10(2):e0004386.

## Supporting information

S1 Fig Flowchart of article screening process and reasons for exclusion of articles. (DOCX)
