# Supplementary material for: Soil-transmitted helminths: A critical review of the impact of co-infections and implications for control and elimination
Source: PLoS Negl Trop Dis. 2023 Aug 10;17(8):e0011496. doi: 10.1371/journal.pntd.0011496 (PMC10414660; doi:10.1371/journal.pntd.0011496)
Supplement: S1 Fig — (DOCX) [file pntd.0011496.s001.docx]

**Soil-transmitted helminths: a critical review of the impact of co-infections and implications for control and elimination**

**SUPPORTING INFORMATION**


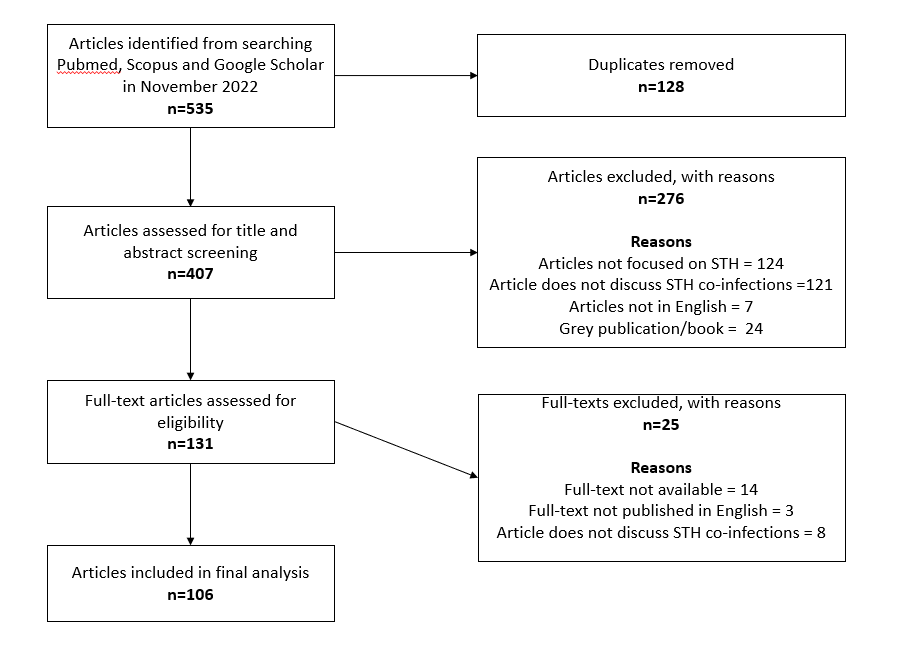


S1 Fig. Flowchart of article screening process and reasons for exclusion of articles
